# Intraocular solitary extramedullary plasmacytoma presenting as unilateral anterior and intermediate uveitis preceded by refractory glaucoma

**DOI:** 10.1186/s12886-021-01822-9

**Published:** 2021-01-30

**Authors:** Tom Ayton, Svetlana Cherepanoff, David Gottlieb, William A. Sewell, Sandy Smith, Claire Hooper

**Affiliations:** 1 University of New South Wales, Sydney, Australia; 2 St Vincent’s Hospital, Sydney, Australia; 3 Westmead Hospital, Sydney, Australia; 4 University of Sydney, Sydney, Australia; 5 Garvan Institute of Medical Research, Sydney, Australia; 6 Save Sight Institute , Sydney, Australia

**Keywords:** Solitary Extramedullary Plasmacytoma, Intraocular, Uveitis, Refractory Glaucoma

## Abstract

**Background:**

Solitary extramedullary plasmacytoma (SEP) is a localised proliferation of monoclonal plasma cells involving soft tissue with no or minimal bone marrow involvement and no other systemic evidence of multiple myeloma. Intraocular involvement is exceedingly rare.

**Case presentation:**

We report a 78-year-old man who was referred with glaucoma in the right eye. He subsequently developed anterior chamber (AC) inflammation and refractory glaucoma then dense vitritis. A vitrectomy was performed with the biopsy revealing numerous plasma cells with atypical findings. In conjunction with the flow cytometry results, and a systemic work up excluding multiple myeloma, a diagnosis of SEP was made. The patient was treated with ocular external beam radiotherapy with resolution of the intraocular inflammation and control of the intraocular pressure. He remains well with no local recurrence and no development of multiple myeloma over a follow up period of 2.5 years.

**Conclusions:**

This is the first case report of SEP presenting as intraocular inflammation without a uveal tract mass.

## Background

Solitary plasmacytomas are part of the spectrum of plasma cell neoplasms. By definition, they remain localised with no or minimal bone marrow infiltration by clonal plasma cells (plasma cells < 10%) and no other evidence of systemic spread [1]. However, they do have a propensity to eventually progress to multiple myeloma, to which they are cytologically and immunophenotypically very similar. They represent around 5% of plasma cell dyscrasias and are rare with a cumulative incidence of 0.15/100,000 and a slight male preponderance [1]. Solitary plasmacytomas are further subtyped into solitary bone plasmacytomas and solitary extramedullary plasmacytomas. Approximately 80% of SEPs occur in the upper aerodigestive tract, particularly the paranasal sinuses, pharynx and tonsil, but they can involve any site or organ [2].

Orbital and adnexal extramedullary plasmacytomas are rare with most cases occurring in the context of pre-existing multiple myeloma [3]. Uveal involvement by plasma cell neoplasms is exceedingly rare with only eight cases reported in the literature, of which only two were SEP, and one case report describing a monoclonal gammopathy of unknown significance presenting as vitritis and retinal vasculitis [4–8]. We describe herein a case of intraocular SEP presenting as anterior uveitis and dense vitritis with no uveal tract mass seen clinically or on imaging.

## Case presentation

A 78-year-old man was referred in 2008 with mildly elevated right intraocular pressure (IOP) and optic disc damage. Best corrected visual acuities (BCVA) were 6/6–2, N5 bilaterally. His IOPs were 24 mmHg right and 21 mmHg left with cup-to-disc ratios (CDR) of 0.75 right and 0.45 left. He was commenced on g latanoprost nocte initially in the right eye and later switched to g travoprost/timolol nocte in both eyes. Six years later, his BCVAs were 6/6 bilaterally, IOPs were 21 mmHg right and 22 mmHg left and the CDRs were unchanged. In January 2015, he underwent uneventful left cataract surgery. Two months later, he presented with blurred right vision. Right BCVA was 6/60, IOP 52 mmHg and CDR 0.85. Left vision was 6/6 unaided and IOP was 12 mmHg. The right IOP was controlled with oral acetazolamide 250 mg BD in addition to g travoprost/timolol nocte in both eyes. One week later, his right BCVA had improved to 6/18 but he was found to have 3+ cells in the right AC and 1+ cells in the left AC. There was no iris mass and gonioscopy revealed an open drainage angle. There was no vitritis or posterior segment involvement. He was commenced on g prednisolone acetate hourly which was tapered to QID in both eyes within two weeks. Baseline uveitis blood tests and chest Xray were normal.

The left AC inflammation settled quickly but the right eye continued to require g prednisolone acetate QID to keep the AC quiet. The right IOP could not be controlled medically so he underwent right trabeculectomy with mitomycin C five months later. He required four needlings with 5-fluorouracil (5-FU) to maintain the bleb. G prednisolone acetate was tapered and ceased in February 2016. In May 2016, his right BCVA was 6/60 and the left was 6/6 unaided with IOPs of 14 mmHg right and 18 mmHg left on g travoprost/timolol nocte in the left eye. The view of the right posterior pole was hazy due to cataract but there were no AC cells or vitritis. The right AC inflammation recurred in August 2016 and he was recommenced on g prednisolone acetate and required a further two needlings of the bleb with 5-FU. He underwent right cataract surgery in November 2016 and was noted to have vitreous opacities post-operatively with right BCVA of 6/60. In March 2017, his right BCVA dropped to hand movements and he underwent a vitrectomy with a 1 ml undiluted and 5 ml diluted sample, as well as the vitreous cassette, sent to pathology.

**Fig. 1:**
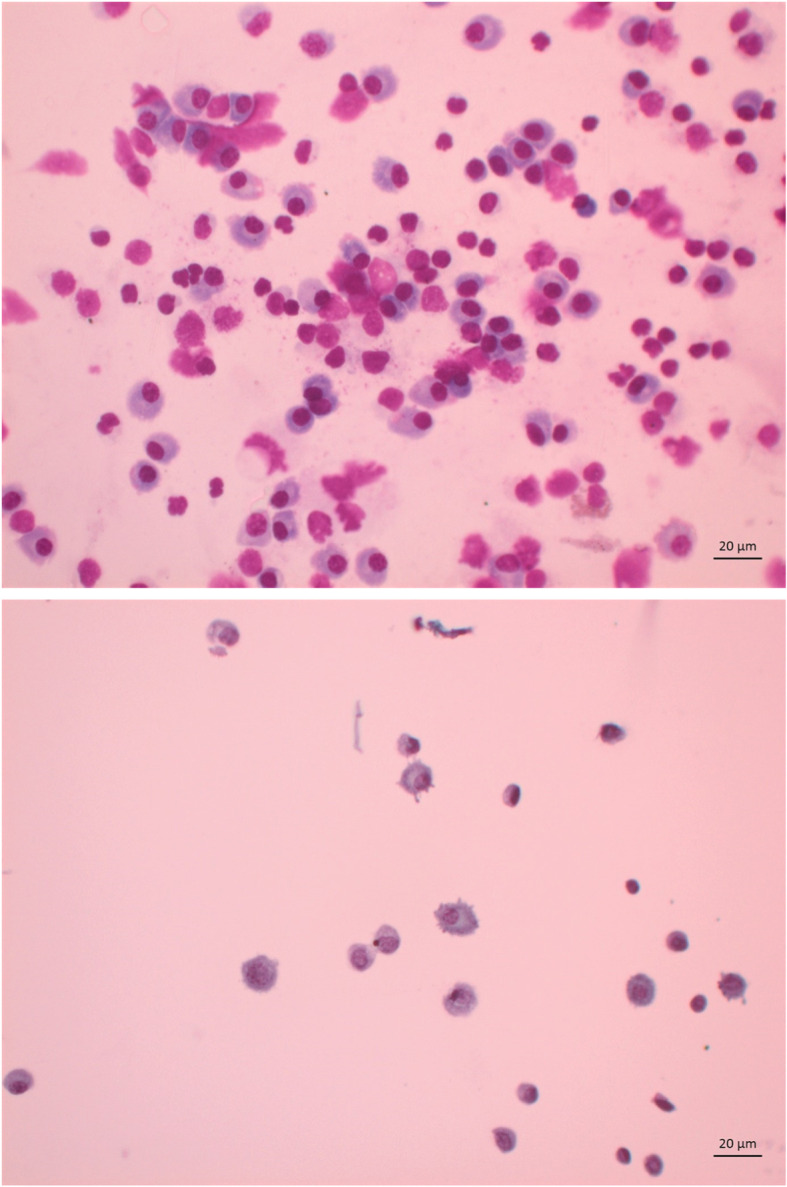
Vitreous sample of the right eye. Numerous plasma cells with atypical findings including binucleation, nuclear pleomorphism and conspicuous nucleoli

**Fig. 2:**
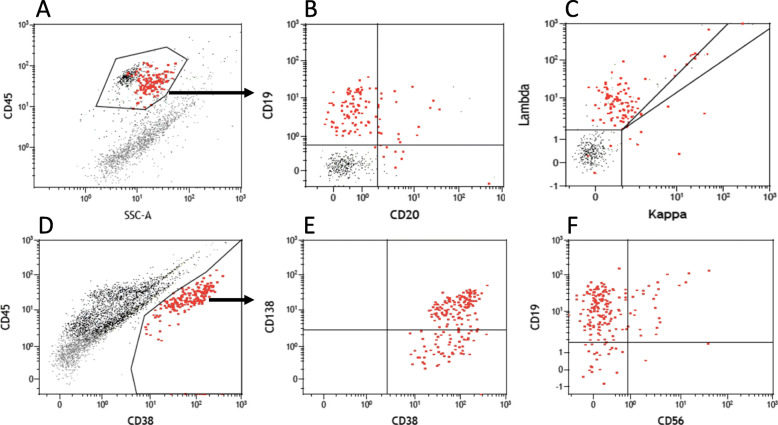
Flow cytometry of vitreous sample. The plasma cells are shown in red and other cells in black. The analyses in Panels **a**-**c** were completed 3 h after sample collection, and the analyses in Panels **d**-**f** were completed 24 h later. 2000–4000 total events were acquired in each tube. Event numbers were limited by the cells present in the available sample. Plasma cells were approximately 5% of events. Samples were analyzed on a FACSCanto II (Becton Dickinson, San Jose, CA). Events were acquired in FACSDiva software (BD) and analyzed in Kaluza software (Beckman Coulter, Brea, CA). Panels A-C: Cells were stained with a cocktail of CD45-V500, CD19-V450, κ-FITC, λ-PE, CD20-PerCP, CD10-PE-Cy7, CD11c-APC and CD3-APC-H7 as described (Tran DN et al. 2016). **a**: All cells are shown. The plasma cells have moderate side scatter and are moderately bright for CD45. **b** and **c**: only the cells in the gate in Panel **a** are shown. **b**: The plasma cells are CD19+ and CD20-. **c**: The plasma cells are surface lambda positive. Panels **d**-**f** were stained with CD45-V500, CD38-V450, intracellular κ-FITC, intracellular λ-PE,CD20-PerCP, CD56-PE-Cy7, CD138-APC and CD19-APC-H7 as described (Tran et al. 2016). **d**: All cells are shown. The plasma cells are very bright for CD38. **e** and **f**: only the cells in the gate in Panel **d** are shown. **e**: The plasma cells are substantially CD138+. **f**: The plasma cells are CD56 negative

The cytospins were highly cellular with numerous plasma cells, some with atypical findings including binucleation, nuclear pleomorphism and conspicuous nucleoli, in addition to small to intermediate sized lymphocytes and eosinophils (shown in Fig. 1). Flow cytometry demonstrated a clonal lambda-restricted population of cells with a phenotype largely consistent with plasma cells, except that the lambda positivity was detectable by surface staining (shown in Fig. 2), whereas aberrant or monoclonal plasma cells are typically negative for light chains by surface staining. All of the plasma cells had high forward scatter, moderate side scatter and were positive for CD45. The cells were CD38 positive (very bright), CD19 positive (moderately bright), CD22 positive and CD138 positive. CD5, CD10, CD11c, CD20, CD23, CD56 and CD200 were negative. Flow cytometry also demonstrated normal T cells, but there were no normal or lymphomatous B cells. The plasma cells were more abundant in the cytology than in the flow cytometry. It has been well documented in bone marrow aspirates that flow cytometry under-estimates the abundance of plasma cells, but nevertheless is reliable in detecting monoclonal populations [9]. There were no polymorphs or organisms on the Gram Stain of the vitreous sample. No anaerobes or fungi were isolated. Adenovirus and enterovirus ribonucleic acid and herpes simplex virus and varicella zoster virus were negative by polymerase chain reaction.

**Fig. 3:**
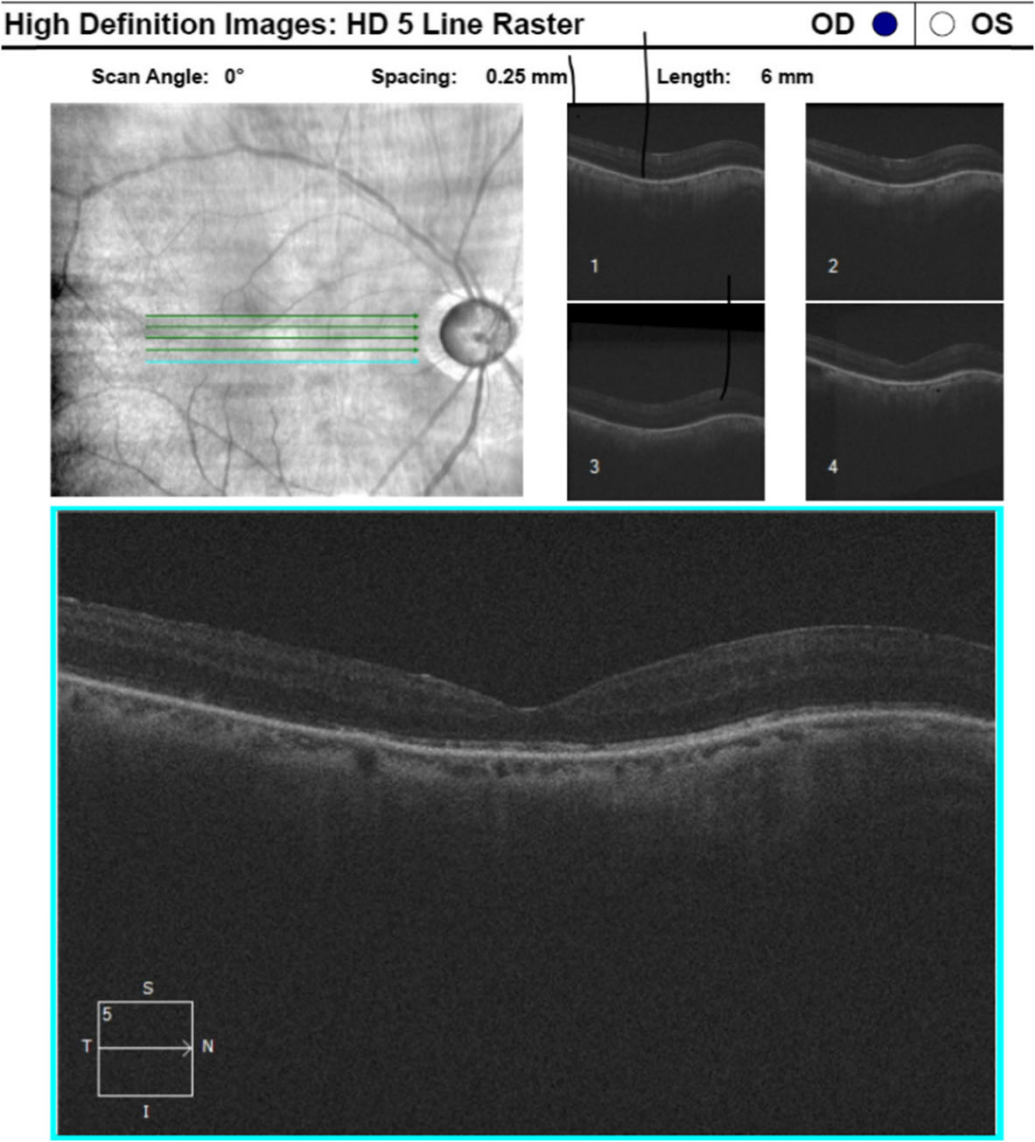
OCT Macula of the right eye. Thin choroid consistent with the patient’s age. No subretinal deposits

One-week post vitrectomy, the right IOP was 7 mmHg and there were 3+ cells in the right AC on g prednisolone acetate qid and g brinzolamide/brimonidine tartrate BD in the right eye. Two weeks later, the right IOP was 34 mmHg and the patient was commenced on g bimatoprost/timolol RE and oral acetazolamide 250 mg TDS. There were no choroidal elevations, deposits or pigmentary change on examination. Ocular coherence tomography was normal with a thin choroid (111 μm right eye and 107 μm left eye) on enhanced depth imaging (shown in Fig. 3).

The patient was referred to a haematologist. Work-up for systemic disease was unremarkable. The only finding was a very small monoclonal B cell lymphocytosis (MBL) population of lambda positive B cells in the peripheral blood, comprising 0.5% of lymphocytes, with an absolute clone size of 0.01 × 10^9^/L. These cells were positive for CD5, CD19, CD20 and CD23. Serum immunofixation was negative. Serum free light chains were normal. Bone marrow biopsy showed normal morphology and flow cytometry with no evidence of lymphoma or multiple myeloma, and specifically no evidence of the plasma cells found in the vitreous sample. Positron emission tomography-computed tomography (PET-CT) imaging of the neck, chest, abdomen and pelvis was unremarkable as was an MRI with contrast of the brain and orbits. Cerebrospinal fluid obtained from a lumbar puncture demonstrated no evidence of lymphoma or myeloma by morphology or flow cytometry, with no evidence of the plasma cells found in the vitreous sample.

Ocular external beam radiotherapy of 20Gy in ten fractions of 2Gy was completed in August 2017. Two years later, his unaided distance acuities were 6/60 right and 6/6 left. The right eye had an IOP of 16 mmHg with advanced cupping (CDR 0.99) and the left had an IOP of 13 mmHg with moderate cupping (CDR 0.75) on g brinzolamide/brimonidine tartrate BD and g brimatoprost/timolol nocte in both eyes and oral acetazolamide 250 mg BD. The AC and vitreous were quiet in both eyes. The most recent correspondence from the patient’s haematologist revealed no evidence of development of multiple myeloma 2.5 years later.

## Discussion and conclusion

The diagnosis of SEP requires histological and immunohistochemical confirmation of the presence of a homogenous infiltrate with monoclonal plasma cells, which typically express CD138 and/or CD38 [1]. The vitreous cells from our patient expressed both CD138 and CD38 and monoclonality was demonstrated by lambda light chain restriction. However, the cells were unusual in that they had features characteristic of normal plasma cells, being moderately bright CD45 positive, very bright CD38 positive, CD19 positive and CD56 negative. By contrast, in most cases of multiple myeloma, the plasma cells are dim or negative for CD45, bright (but not very bright) CD38, CD19 negative and, in around two thirds of cases, CD56 positive [10]. However in SEP, the monoclonal plasma cells can be CD45 positive, CD19 positive, CD56 negative and surface immunoglobulin positive, a phenotype more consistent with polyclonal plasma cells than with multiple myeloma [10, 11]. The possibility of plasmablastic lymphoma was considered, but was thought very unlikely because morphology was not consistent with immunoblasts but with more mature plasmacytoid cells. In addition, the patient was neither immunocompromised by HIV or exogenous immune suppression. Although these are not essential for the diagnosis of plasmablastic lymphoma, PBL is almost always an aggressive malignancy and in addition to the morphology, the subsequent benign clinical course would not support that diagnosis.

The flow cytometry phenotype seen in our patient of CD45 positive, CD19 positive, CD38 very bright positive and CD56 negative can be found in the monoclonal plasma cells in cases of lymphoplasmacytic lymphoma (Waldenstrom’s macroglobulinemia) [11]. However, there was no monoclonal B cell population in either blood or bone marrow or serum IgM paraprotein, so the diagnosis of lymphoplasmacytic lymphoma was considered unlikely. A Mucosa-Associated Lymphoid Tissue lymphoma (MALToma) with plasmacytic differentiation of lymphoid cells was also considered. Although our case was CD20 negative, typical MALTomas are CD20 positive B cells [12]. However, some cases show plasmacytic differentiation, and it has been argued that extraosseous plasmacytomas may represent plasmacytic differentiation of marginal zone lymphoma [13]. Regardless of the cell of origin, when the morphology and flow were taken together, plasmacytoma was considered the most likely diagnosis.

The systemic work up was unremarkable aside from a very small population of CD5, CD19, CD20 and CD23 positive lambda expressing B cells in the peripheral blood. These were not identified in the bone marrow, the vitreous sample or the CSF, and likely represent a small monoclonal B cell lymphocytosis. It was considered an incidental finding as the cells in the vitreous sample were negative for CD5, CD20 and CD23, and therefore phenotypically distinct from the peripheral blood population. MBL populations are common in older individuals, and in one study were found in 3.8% of healthy individuals over the age of 65 [14]. In SEP, the lack of monoclonal plasma cells in the bone marrow may be a good prognostic factor. In a study of 29 patients with SEP followed for a median of three years, 6% with negative bone marrow by flow cytometry progressed to multiple myeloma compared with 20% of those with positive bone marrow by flow cytometry [15].

Two other cases of uveal SEP have been described. An asymptomatic 76-year-old woman was found to have two amelanotic choroidal lesions. Histology and immunocytochemical studies confirmed a plasmacytoma. Serum electrophoresis revealed an immunoglobulin kappa spike but, following ocular external beam radiotherapy, she remained free of local recurrence and did not develop multiple myeloma over a follow up period of nine years [5]. A 55-year-old woman presented with acute anterior uveitis refractory to topical corticosteroids and then developed an iridociliary mass one month later. Plasmacytoma was diagnosed by fine needle aspiration biopsy and the eye was treated with plaque radiotherapy. Two years later, the fellow eye presented with acute anterior uveitis and an iridociliary mass. Plasmacytoma was again diagnosed on biopsy and the eye was treated with plaque radiotherapy. A complete systemic work up performed on both occasions was unremarkable. She remained free of local recurrence and did not develop multiple myeloma over a follow up period of three years [6].

A 67-year-old man also presented with acute anterior uveitis, raised IOP of 42 mmHg and was found to have right iris thickening but he had a pre-existing diagnosis of IgG kappa multiple myeloma [8]. No iris or iridiocilary mass was seen in our case on anterior segment examination or gonioscopy but could not be definitely ruled out as we did not have access to ultrasound biomicroscopy. The refractory glaucoma was most likely due to plasma cells infiltrating the trabecular meshwork [4]. One other case of intraocular inflammation without a uveal tract mass was identified in the literature but this was in the context of a diagnosis of monoclonal gammopathy of unknown significance (MGUS). A 61-year-old man presented with a 3-month history of worsening floaters and was found to have vitritis and retinal vasculitis. A vitreous biopsy was not performed but serum protein electrophoresis resulted in a diagnosis of MGUS which progressed to multiple myeloma three years later [7].

Localised radiotherapy is the mainstay of treatment for SEP which is highly radiosensitive. Local response rates exceed 80–90% particularly if there is no bulky tumour [1]. However, there is a propensity for solitary plasmacytomas to progress to multiple myeloma at a median time of two years. The risk of progression is greater for solitary bone plasmacytoma than SEP and for cases with minimal rather than no bone marrow involvement [1]. The serum free light chain ratio is also an independent risk factor for the progression of solitary plasmacytoma to multiple myeloma when it is outside the normal range [1]. Our patient underwent laboratory evaluation every three months for the first two years and is now having six-monthly reviews. A PET-CT was performed at three months after completion of radiation and then every six months thereafter. To date, he has not developed local recurrence or multiple myeloma over a follow up period of 2.5 years.

## Data Availability

The datasets used and/or analysed during the current study available from the corresponding author on reasonable request.

## Abbreviations

SEP: Solitary extramedullary plasmacytoma
AC: Anterior chamber
IOP: Intraocular pressure
BCVA: Best corrected visual acuity
CDR: Cup-to-disc ratios
5-FU: 5-fluorouracil
MALToma: Mucosa-associated lymphoid tissue lymphoma
MGUS: Monoclonal gammopathy of unknown significance
PET-CT: Positron emission tomography-computed tomography
